# Comparative Study on Immune Function of the Head and Trunk Kidney in Rainbow Trout Responding to IHNV Infection

**DOI:** 10.3390/v14122663

**Published:** 2022-11-28

**Authors:** Ruhan Sun, Qin Wang, Zhenyu Huang, Mengting Zhan, Zhangchun Zhao, Bingchao Wang, Mengge Guo, Le Yuan, Zechao Shi, Gang Ouyang, Wei Ji

**Affiliations:** 1 Department of Aquatic Animal Medicines, College of Fisheries, Key Laboratory of Freshwater Animal Breeding, Ministry of Agriculture and Rural Affair/Key Laboratory of Agricultural Animal Genetics, Breeding and Reproduction, Ministry of Education, Huazhong Agricultural University, Wuhan 430070, China; 2 Key Laboratory of Freshwater Ecology and Biotechnology, Institute of Hydrobiology, Chinese Academy of Sciences, Wuhan 430072, China; 3 The Key Laboratory of Aquaculture Disease Control, Ministry of Agriculture, Wuhan 430072, China; 4 Yangtze River Fisheries Research Institute, Chinese Academy of Fishery Sciences, Wuhan 430223, China

**Keywords:** head kidney, trunk kidney, immune, IHNV

## Abstract

A teleost’s kidney was divided into head kidney and trunk kidney. The head kidney is an important lymphatic organ, while the trunk kidney mainly performs osmotic pressure regulation and excretion functions. Previous studies have shown that the teleost’s head kidney exerts a strong immune response against pathogen invasion, while the mechanism of immune response in the trunk kidney is still rarely reported. Therefore, in this study, we established an Infectious hematopoietic necrosis virus (IHNV) immersion infection model to compare the similarities and differences of immune response mechanisms between the head kidney and trunk kidney against viral infection. The results showed that IHNV infection causes severe tissue damage and inflammatory reaction in the head and trunk kidney, triggers a series of interferon cascade reactions, and produces strong immune response. In addition, the transcriptome data showed that the head kidney and trunk kidney had similar immune response mechanisms, which showed that the NOD-like receptor signaling pathway and Toll-like receptor signaling pathway were activated. In conclusion, despite functional differentiation, the teleost’s trunk kidney still has a strong immune response, especially the interferon-stimulated genes, which have stronger immune response in the trunk kidney than in the head kidney when responding to IHNV infection. This study contributes to a more comprehensive understanding of the teleost immune system and enriches the theory of kidney immunity in teleosts.

## 1. Introduction

Compared to mammals, fish lack bone marrow and lymph nodes, and instead the kidney is the main lymphatic organ in teleosts besides the thymus, spleen, and mucosa-associated lymphatic tissue [1]. The teleost’s kidney originates in the mesoderm, and in early life, the kidney exercises excretion functions; then, during development the kidney differentiates into head kidney (also named anterior kidney) and trunk kidney (also middle kidney) [2]. The head kidney resembles the mammalian adrenal gland and is an important endocrine and hematopoietic organ in teleost fish [3,4]. The trunk kidney is a unique organ of the teleost, which fulfils the functions of excretion and the osmoregulation of water and salt [5].

Structural differences between the head and trunk kidney lead to their functional differentiation. The head kidney consists of a reticular network of stromal cells and groups of haemopoietic cells [6,7], and the stromal is often called reticuloendothelial stromal, which consists of endothelial cells, outer membrane cells covering the outer surface of endothelial cells, and phagocytic reticular cells [8], while the trunk kidney consists of glomeruli, renal tubules, and hematopoietic tissues [9]. There are also significant differences in the kidney morphology of different fishes; the kidneys of some marine fishes do not contain renal corpuscles and are called glomerular kidneys [9]. The kidney is an important tissue for processing pathogens, and much research focuses on head kidney mediated immune responses in teleosts [10]. Through transcriptome sequencing, Chen et al. found that a large number of immune-related genes were significantly expressed in the head kidney of grass carp after grass carp reovirus (GCRV) infection [11]. In addition, Chettri et al. found that stimulation with different pathogen-associated molecular patterns (PAMPs) could induce strong immune response in head kidney leukocytes of rainbow trout [12]. However, the immune mechanism of the trunk kidney is rarely reported, and the comparison of immune response between head kidney and trunk kidney has not been reported.

Infectious hematopoietic necrosis virus (IHNV), which belongs to the genus *Rhabdovirus* of the *Rhabdoviridae* family, is susceptible to most salmonids and can cause mortality rates as high as 90% or more [13,14]. IHNV infection can cause necrosis of the hematopoietic organs of fish, and cause pathological features such as abdominal swelling, proptosis, caudal fin hemorrhage, anus redness and swelling, and pale gill filaments [15]. Our previous studies showed that IHNV infection of the brain and intestine of rainbow trout would trigger a strong immune response, causing disorder of the blood–brain barrier (BBB) and imbalance of the intestinal flora [16,17]. In addition, Yu et al. showed that air-filled organs (swim bladders) can produce a series of mucosal immune responses mediated by B cells after IHNV infection [18]. Although these experimental data indicate that IHNV can induce strong immune response in rainbow trout, the response mechanism of the head and trunk kidney after IHNV infection is still unclear.

In order to compare the immune response mechanisms of the head and trunk kidney after IHNV infection, we established a model of IHNV immersion infection in rainbow trout, and higher levels of IHNV load were found in the head and trunk kidney at 7 days post-infection (dpi). Our data indicated that IHNV infection causes severe tissue lesions and inflammatory reactions in the head and trunk kidney, triggers a series of interferon cascade reactions, and produces strong immune response. In addition, transcriptome analysis showed that the head and trunk kidney produced similar immune response mechanisms, which triggered the Toll-like receptor (TLR) and NOD-like receptor (NLR) signaling pathways. Interestingly, after IHNV infection, the expression of some immune genes, such as interferon-stimulated genes (ISGs), was higher in the trunk kidney than in the head kidney. Our study contributes to a more comprehensive understanding of the teleost immune system and enriches the theory of fish kidney immunity.

## 2. Materials and Methods

### 2.1. Experimental Fish

Rainbow trout, with a body weight of 10–15 g, were obtained from a fish farm in Shiyan, Hubei Province. And the fish were maintained in water tanks controlled by a circulating water filtration system with water temperature of 15 °C. The rainbow trout was fed with commercial trout pellets at a rate of 0.5–1% biomass twice a day. All fish stopped being fed 48 h prior to sampling. This experiment was carried out under the approval of the Animal Experiment Committee of Huazhong Agricultural University.

### 2.2. Challenging with Infectious Hematopoietic Necrosis Virus (IHNV)

The cyprinus carpio epithelioma papillosum cyprini (EPC) cell line was maintained in minimum Eagle’s medium (MEM) supplemented with 10% fetal bovine serum (FBS) (Viva cell, Shanghai, China) and 1% penicillin–streptomycin solution, and cultured in an incubator at 16 °C. The virus was passed in vitro by EPC cell line, titration, and adjusted to 1 × 10^9^ PFU ML^−1^ in MEM, then stored at −80 °C for later use. The IHNV was gifted from Dr. Hong Liu (Shenzhen Entry-Exit Inspection and Quarantine Bureau). For challenging experiments, the fish were bathed in 10 L aeration water containing 1 mL of IHNV suspension (1 × 10^9^ pfu/mL), and then transferred to a new tank with aquatic water. The control fish were maintained in a similar tank and exposed to MEM without IHNV.

### 2.3. Sampling

For sampling, fish were anesthetized with MS-222, and tissues were collected at the indicated time points after infection. For histological and pathological studies, the head and trunk kidney of rainbow trout were removed from control and infected fish, and immediately fixed in 4% (*v*/*v*) neutral buffer solution paraformaldehyde for more than 24 h. For RNA extraction and real-time fluorescent quantitative PCR (qRT-PCR), the head and trunk kidney were stored in Trizol reagent at −80 °C. 

### 2.4. RNA Isolation and qRT-PCR Analysis

Total RNA was extracted from different tissues using Trizol Reagent (Invitrogen, Carlsbad, CA, USA) according to the manufacturer’s instructions. The extracted RNA was quantified by spectrophotometry (NanoPhotometer NP 80 Touch, Implen, Germany) and the integrity of RNA was detected by 1% agarose gel electrophoresis. Then, the extracted RNA was reverse transcribed into cDNA using Hifair III 1st Strand cDNA Synthesis SuperMix for qPCR (YEASEN, Shanghai, China), according to the manufacturer’s instructions. The synthesized cDNA was diluted 3 times to 300 ng/μL and then was used as a template for qRT-PCR analysis. The qRT-PCR was performed on a qTOWER3G PCR system (Analytik Jena AG, Germany) under the following conditions: 95 °C for 5 min, followed by 40 cycles at 95 °C for 10 s and at 58 °C for 30 s. The 2^−ΔΔCt^ method was used to calculate the fold changes of gene expression, and EF-1α was used as an internal reference gene.

### 2.5. Histology and Light Microscopy Studies

The trunk and head kidney were taken from rainbow trout at different infection time points and immediately immersed in 4% neutral formalin buffer for at least 24 h, followed by dehydration in gradient ethanol and washed with xylene. Then the samples were embedded in paraffin, and finally sectioned into pieces of 5 μm. Subsequently, the paraffin sections were stained with hematoxylin and eosin (H&E). Images were acquired with a microscope (Olympus) using the Axiovision software. For the detection of IHNV, paraffin sections were incubated at 4 °C overnight with mouse anti-IHNV (0.2 μg/mL), then incubated with secondary antibody (Cy3-AffiniPure goat anti-mouse IgG, 1.5 μg/mL) at room temperature for 40 min. Finally, images were obtained under a fluorescence microscope (Olympus, Tokyo, Japan) using Axovision software. The parameters of each image were measured by three different researchers and averaged to reduce random errors.

### 2.6. Standard Curve for IHNV

The recombinant plasmid was constructed using PCR products of IHNV-G fragment after appropriate modifications as described previously [19]. Plasmid DNA was isolated from an overnight selective culture using the HiPure Plasmid Micro Kit (OMEGA Bio tek). The recombinant plasmids diluted 10 times continuously (from 9.94 × 10^7^ copies/μL~ 9.94 × 10^−1^ copies/μL) were used as the standard positive template. The standard curve is shown in Figure S1, and the Ct values of the samples were extrapolated into the standard curve to calculate the copy numbers. 

## 3. Results

### 3.1. Successful Construction of the IHNV Infection Model in Rainbow Trout

**Figure 1:**
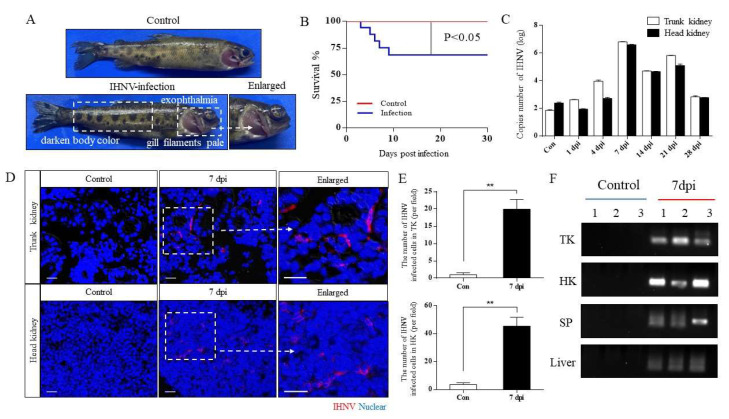
Successful construction of IHNV infection model of rainbow trout. (**A**) The phenotype of rainbow trout after IHNV infection. The top and bottom were the control and infected groups, respectively. (**B**) The survival rate of rainbow trout after IHNV infection. (**C**) The copy number of IHNV in the head and trunk kidney was detected in the control and infected rainbow trout at 1, 4, 7, 14, 21, and 28 dpi (*n* = 6 fish per group). (**D**) Histological examination of IHNV signal in the head and trunk kidney of the control and infected group at 7 dpi, with merged immunofluorescence staining with IHNV (red) and nuclei (blue). Scale bar, 20 μm. (**E**) Statistical results of immunofluorescence IHNV signal (*n* = 6 fish per group). (**F**) IHNV load of trunk kidney (TK), head kidney (HK), spleen (SP), and liver at 7 dpi were detected by PCR. ** *p* < 0.01 (unpaired Student’s *t* test).

In order to explore the similarities and differences of immune responses between the head kidney and trunk kidney responding to virus infection, we established an IHNV immersion infection model. In the infection group, the rainbow trout showed obvious pathological changes, such as exophthalmia, gill filaments pale, darken body color, and caudal fin hemorrhage (Figure 1A). The survival rate of the infection group was stable at about 68%, which proved that the infection concentration was appropriate (Figure 1B). In addition, to further determine the changes of virus load over time, we measured the number of IHNV copies in the head and trunk kidney. As a result, IHNV copies have similar change trends in the head and trunk kidney, and the highest IHNV copies were detected in the trunk and head kidney at 7 dpi (~6.6 × 10^6^, 4.1 × 10^6^), and then the IHNV copies gradually decreased and returned to the level of control group at 28 dpi (Figure 1C). Through immunofluorescence, the IHNV-infected cells were detected in the head and trunk kidney, and the number of IHNV infected cells was significantly increased after IHNV infection (Figure 1D,E). Moreover, agarose gel electrophoresis further confirmed that IHNV invaded the head and trunk kidney as well as the spleen and liver at 7 dpi, and no positive bands were observed in the control group (Figure 1F). Overall, these results indicated that the IHNV infection model was successfully established, and the virus load in the head and trunk kidney showed similar change trends.

### 3.2. IHNV Infection Causes Pathological Changes in Rainbow Trout Kidney

**Figure 2:**
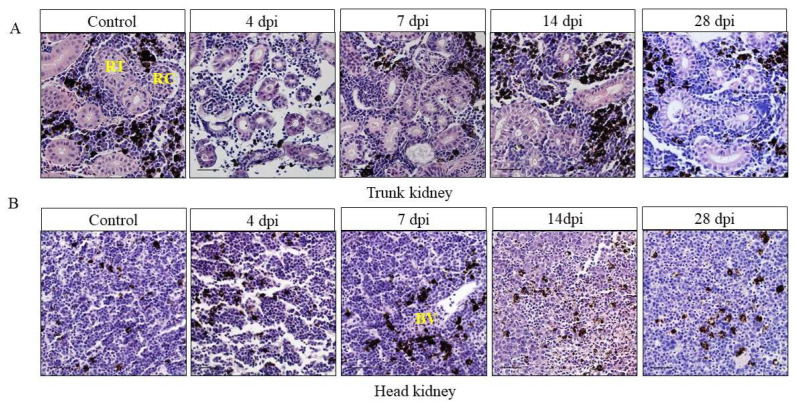
Pathological changes in the trunk kidney (**A**) and head kidney (**B**) of rainbow trout after infection with IHNV. After IHNV infection, rainbow trout kidney showed significant pathological changes in the infection group at 4, 7, 14, and 28 dpi compared to the control group (H&E). RT: renal tubular; RC: renal corpuscle; BV: blood vessel. Scale bar, 20 μm.

In order to clarify the histological organization of the head and trunk kidney, and whether pathological changes occurred after IHNV infection, H&E staining was performed on paraffin sections of the head and trunk kidney at different infection time points. The trunk kidney is composed of renal tubules (RT), renal corpuscle (RC), and hematopoietic tissue, while the head kidney lacks the nephron, which is composed of a mesh matrix cells that support different hematopoietic populations and contains a relatively rich blood vessel (BV) (Figure S2). Through H&E staining, we found that serious pathological changes occurred in the trunk kidney at 4 and 7 dpi, which were manifested as necrosis and shedding of renal tubular epithelial cells, necrosis of renal corpuscles, glomerular enlargement, and melanin macrophages decrease. Then the histological morphology gradually recovered and was comparable to the control group at 28 dpi (Figure 2A). Interestingly, similar changes occurred in the head kidney, the serious pathological changes occurred at 4 and 7 dpi, such as lymphatic disorder, vascular fluid, and bleeding point, and the histological morphology gradually recovered at 28 dpi (Figure 2B). Additionally, we also scored the degree of kidney lesions. According to the score, the pathological changes of the head and trunk kidney were most obvious at 4 and 7 dpi (Tables S1 and S2). All these results indicated that the head kidney and trunk kidney have different structures and IHNV infection can destroy both the structure of the head and trunk kidney.

### 3.3. IHNV Infection Induces the Expression of Immune Genes in the Kidney of Rainbow Trout

**Figure 3:**
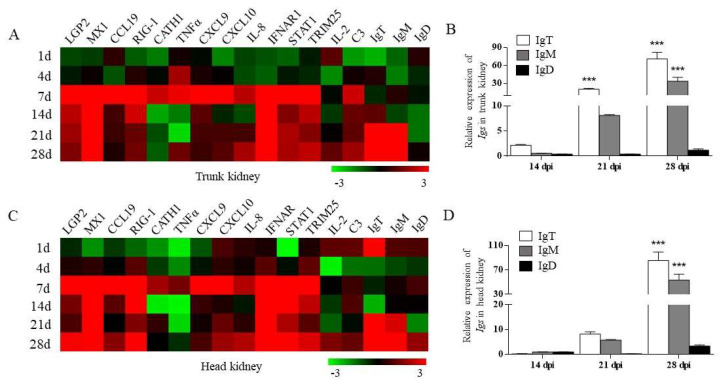
The relative mRNA expression of immune-related genes in the trout kidney after IHNV infection. (**A**,**C**) Heatmap results from qRT-PCR analysis for selected immune-related genes in trunk kidney (**A**) and head kidney (**C**) at 1, 4, 7, 14, 21, and 28 dpi (*n* = 6 fish per group). (**B**,**D**) Relative mRNA expression of IgT, IgM, and IgD at 14, 21, and 28 dpi in rainbow trout trunk kidney (**B**) and head kidney (**D**) (*n* = 6 fish per group). Data are representative of three independent experiments (mean ± SEM). * *p* < 0.05, ** *p* < 0.01, *** *p* < 0.001 (unpaired Student’s *t*-test).

In order to study the expression level of immune-related genes in rainbow trout kidney after IHNV infection, we detected 17 immune-related genes by qRT-PCR, including antiviral genes (LGP2, MX1, TRIM25, RIG-1, TNFα, CATH1, IFNAR1, STAT1), pro-inflammatory cytokine genes (IL-8, IL-2, CCL19, CXCL9, CXCL10), complement factors (C3) and Ig heavy chain genes (IgT, IgM, and IgD). We found that most immune-related genes (antiviral genes and inflammatory genes) showed similar trends in the head and trunk kidney, with significant upregulation at 7 dpi, but interestingly, the expression of TNFα in the trunk kidney was significantly higher than that in the head kidney (Figure 3A,C). In addition, the mRNA expression of IgT (∼60-fold) and IgM (∼30- fold) in the trunk kidney, and IgT(~70-fold) and IgM(~40-fold) in the head kidney was upregulated significantly at 28 dpi (Figure 3B,D).

### 3.4. Kinetics of Immune Response in Head and Trunk Kidney after IHNV Infection

We further analyzed the differences and similarities of dynamic immune responses of head and trunk kidney in rainbow trout after IHNV infection by RNA-seq. According to the expression profiles of immune-related genes, we selected samples at 7 and 28 dpi for RNA-seq and further comparative analysis with control samples. 

**Figure 4:**
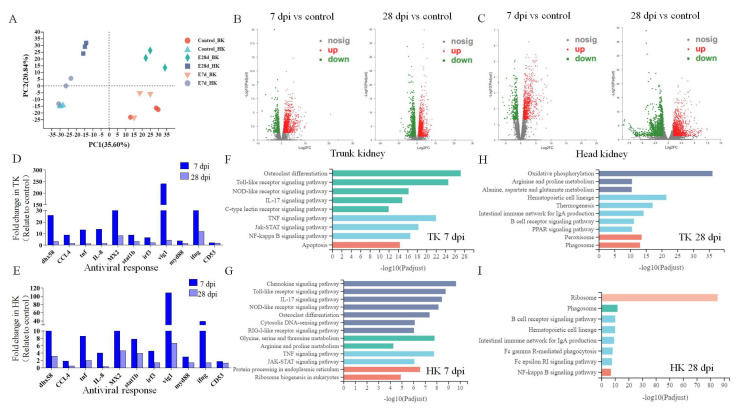
Transcriptome analysis was performed to analyze the immune response dynamics of trunk and head kidney of rainbow trout after IHNV infection. (*n* = 3 fish per group.) (**A**) PCA (principal component analysis) cluster plots of gene expression levels in different samples. The horizontal axis represents the contribution degree of principal component 1 (PC1) in the two-dimensional graph to the differentiated samples, and the vertical axis represents the contribution degree of principal component 2 (PC2) in the two-dimensional graph to the differentiated samples. The blue, gray, and deep blue represent the head kidney, and the red, green, and orange represent the trunk kidney. Different shapes indicate different groups. (**B**,**C**) Volcano plot displaying the DEGs distribution in the trunk kidney (**B**) and head kidney (**C**) of rainbow trout at 7 dpi (left) and 28 dpi (right) in the infected group compared with control group. Red spots: expression fold change of >2 and FDR of <0.05; green spots: expression fold change of <2 and FDR of <0.05; gray spots: no difference in expression. The horizontal axis represents log2(FC), and the vertical axis represents −log10(FDR). (**D**,**E**) Representative immune-related genes (anti-virus, antigen presentation, and inflammatory) in the trunk kidney (**D**) and head kidney (**E**) modulated by IHNV infection at 7 and 28 dpi. Data are expressed as mean fold increase in expression of these genes based on RNA-seq results. (**F**–**I**) KEGG pathways that were significantly altered in trunk kidney (F and H) and head kidney (**G**,**I**) of rainbow trout at 7 and 28 dpi versus control fish revealed by RNA-Seq. Fold change differences between control and IHNV-infected groups were calculated using cutoff of 2-fold.

Principal component analysis (PCA) is used to detect the consistency between biological replicates and clustering between different groups of samples. PCA plot showed that the head kidney group was separated from the trunk kidney group, and a high level of consistency was present in the biological replicates of the same tissues (Figure 4A). Subsequently, differential expression gene (DEG) analysis was performed. As shown in the volcano diagram, compared with the control group, 1497 and 3297 genes were significantly changed in the trunk kidney at 7 and 28 dpi, among which 1025 and 1604 genes were significantly upregulated and 472 and 1693 genes were significantly downregulated, respectively (Figure 4B). Similarly, 1881 and 3923 genes were significantly changed in the head kidney at 7 and 28 dpi, among which 1357 and 2002 genes were significantly upregulated and 524 and 1921 genes were significantly downregulated, respectively (Figure 4C). Based on these significant DEGs, we selected 11 immune-related genes to analyze their expression levels in the head and trunk kidney after IHNV infection. These immune-related genes included DExH-box helicase 58 (dhx58), C-C motif chemokine ligand 4 (CCL4), myxovirus resistance protein 2 (MX2), interleukin 8 (IL-8), tumor necrosis factor (tnf), signal transducer and activator of transcription 1b (stat1b), interferon regulatory factor 3 (irf3), viperin1 (vig1), myeloid differentiation primary response protein 88 (myd88), interferon gamma (ifng) and CD53 molecule (CD53) (Figure 4D,E). The results showed that most immune-related genes showed similar trends in the head and trunk kidney, with significant upregulation at 7 dpi. Notably, the expression of these immune genes was significantly higher in the trunk kidney than in the head kidney, such as dhx58, MX2 and vig1. In addition, we tested the accuracy of RNA-seq results by qRT-PCR, and randomly selected 6 out of 11 immune-related genes. The results indicated that the transcriptome data were stable and reliable (Figure S3).

### 3.5. Enrichment of KEGG Pathways in the Kidney after IHNV Infection

Subsequently, we performed KEGG functional enrichment analysis on these DEGs to further explore their biological functions (Figure 4F–I). The results showed that in the head and trunk kidney at 7 dpi, the enriched signaling pathways were mostly immune-related signaling pathways, including the TLR signaling pathway, NLR signaling pathway and IL-17-mediated signaling pathway. Interestingly, the RIG-Ⅰ-like receptor signaling pathway was also enriched in the head kidney at 7 dpi, but not in the trunk kidney. At 28 dpi, the signaling pathways enriched in the head kidney were mainly related to cells and molecules, while the signaling pathways enriched in the trunk kidney were mainly related to metabolism.

**Figure 5:**
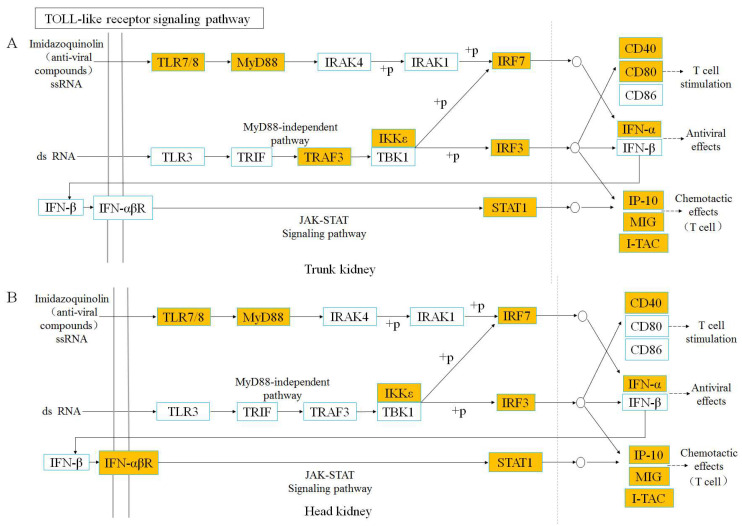
TOLL-like receptor signaling pathway was analyzed and simplified from KEGG. Genes marked in orange represent DEGs in RNA-seq.

**Figure 6:**
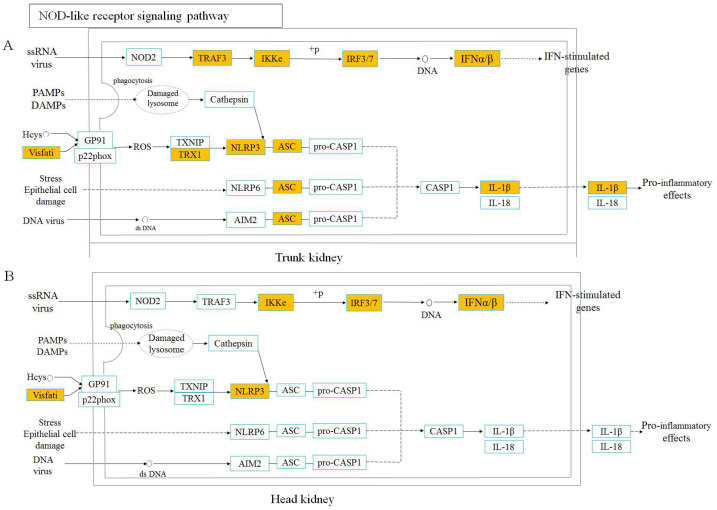
NOD-like receptor signaling pathway was analyzed and simplified from KEGG. Genes marked in orange represent DEGs in RNA-seq.

In order to further explore the similarities and differences of immune response mechanisms between the head and trunk kidney in the process of anti-IHNV infection, we analyzed two pattern recognition receptor signaling (PRRs) pathways (Figure 5 and Figure 6). We found that the key node genes TLR7/8 and MyD88 in the TLR signaling pathway is significantly upregulated in both the head and trunk kidney (Figure S4A), which plays an important role in the detection of the IHNV. In addition, in the NLR signaling pathway, the key node genes IKKε and IFNα/β were also upregulated in the head and trunk kidney (Figure S4B).

## 4. Discussion

In teleosts, the head and trunk kidney have different structures, which results in their main physiological functions being different [2]. The kidney is an important tissue for dealing with pathogens [20]. Most of the previous studies focused on the immune response mediated by the head kidney of teleosts, however, there were few reports on the mechanism of immune response in the trunk kidney. In this study, we systematically compared and elucidated the immune response of the head and trunk kidney in response to virus infection of rainbow trout. 

Firstly, an IHNV immersion infection model was constructed, and after virus infection, rainbow trout showed typical pathological changes, such as eyeball protrusion, gill filaments pale and caudal fin hemorrhage, which are similar to previous studies [15]. In addition, similar viral loads were detected in the head and trunk kidney of rainbow trout, reaching a peak at 7 dpi and returning to control level at 28 dpi, which indicates that the trunk and head kidney have similar virus-clearing abilities. Similar IHNV load was also found in the gallbladder of rainbow trout after IHNV infection, reaching a peak at 4 dpi, and then gradually returning to the control level [21]. Subsequently, we further explored the histopathological changes of the head and trunk kidney after IHNV infection. Interestingly, significant pathological changes were observed in both the head and trunk kidney. In the trunk kidney, glomerular necrosis and tubular epithelial cell necrosis were observed. In the head kidney, there were lymphatic cord disorders, hemorrhagic spots, and other pathological changes. Previous studies have shown extensive bleeding and necrosis in rainbow trout kidney after IHNV infection [22]. In addition, we scored the degree of kidney lesions according to previous studies [23,24,25]. According to the score, the pathological changes of the head and trunk kidney were most obvious at 4 and 7 dpi, which was consistent with the highest viral load at 7 dpi (Tables S1 and S2). In conclusion, our results suggest that the virus replicates in vivo and causes pathological changes in the head and trunk kidney of rainbow trout after IHNV infection. 

Previous studies have shown that pathogen infection can significantly upregulate the expression of immune-related genes in the head kidney [26,27]. However, as the excretion and osmotic pressure-regulating organ of teleosts, there are few reports regarding the immune function of the trunk kidney in processing pathogens [20,28]. In order to compare and analyze the differences and similarities of immune gene expressions in the head and trunk kidney of rainbow trout after IHNV infection, we performed qRT-PCR analysis and most immune genes showed similar expression trends in the head and trunk kidney. Importantly, the mRNA expression levels of signal transducer and activator of transcription 1 (STAT1) were significantly upregulated in the head and trunk kidney at 7, 14, 21, and 28 dpi. And as a key transcription regulator of JAK-STAT signal pathway, STAT1 is widely involved in cytokine mediated signal response and can control IFN-dependent transcriptional activation by activating IFN receptor JAK kinase complexes [29,30]. Previous study has shown that the expression level of STAT1 was also significantly upregulated at 4 dpi in the intestine of rainbow trout after IHNV infection [16]. Thus, the high expression of STAT1 indicates that a strong immune response has been established in the head and trunk kidney. In addition, the interferon (alpha and beta) receptor 1 (IFNAR1) and myxovirus resistance protein 1 (MX1) were also significantly upregulated in the head and trunk kidney after IHNV infection, which medicate innate immune responses and play an important role in resistance to pathogen invasion [31,32,33]. More importantly, as one of the ISGs and the main effector pathways of IFN-mediated antiviral responses, MX1 plays a powerful role in the antiviral response of vertebrates [34]. The study by Shao et al. showed that the expression level of MX1 and other ISGs (ISG15, IFN1, Vig1) were also significantly upregulated in the head kidney of rainbow trout on the 7th day after IHNV infection [35]. In combination with our results, it can be inferred that a similar interferon-mediated innate immune response occurred in the head and trunk kidney of rainbow trout after IHNV infection. Interestingly, our results also showed that the upregulation of the TNFα gene in the trunk kidney was more obvious than that of the head kidney at 7 dpi. As the first member of the TNF superfamily identified in fish, TNFα plays a wide range of biological functions in mediating inflammation, apoptosis, fat metabolism, and organ regeneration [36]. Accumulating data have demonstrated that fish TNFα genes are upregulated during the early stages of infection. After rainbow trout infected with *Yersinia ruckeri* and the viral hemorrhagic septicemia virus (VHSV), the elevation of TNFα transcripts was prominent in the spleen and head kidney [37,38]. In our study, the high expression of TNFα after IHNV infection is involved in the antiviral process of the head and trunk kidney, and the high expression level of TNFα in the trunk kidney may be related to its strong metabolic process [39]. In the present study, the mRNA expression of IgT and IgM were significantly upregulated in the head kidney and trunk kidney at 28 dpi, which indicated that adaptive immune responses mediated by B cells are stimulated in the head and trunk kidney after IHNV infection.

In order to further compare the dynamic changes of immune response between the head and trunk kidney after virus infection, RNA-Seq analysis was performed in the present study. PCA and volcano plot analysis indicated that the changes in gene expression in both the head and trunk kidney were similar at 7 and 28 dpi. We selected 11 representative immune-related genes (dhx58, CCL4, tnf, il-8, MX2, stat1b, irf3, vig1, myd88, ifng, and cd53) from the DEGs to evaluate the transcriptome data. Interestingly, most of the genes had a similar trend in the head and trunk kidney, while the expression level in the trunk kidney was higher than that in head kidney (Figure 4D,E). Among them, Vig1 was significantly upregulated at 7 dpi both in the head and trunk kidney, which is also an ISG, and plays an important role in immune response [40]. After the pathogen invades, recognition of PAMPs by cellular PRRs results in the induction of inflammatory cytokines and IFN genes [41]. Once produced, interferon binds to interferon receptors to induce vig1 expression [42]. Previous studies have shown that vig1 gene is significantly overexpressed in the head kidney of rainbow trout after VHSV infection [43]. In addition to Vig1, ifng was also an ISG and was highly expressed in the head and trunk kidney at 7 dpi.In conclusion, our results suggest that ISGs are activated in the head and trunk kidney after IHNV infection, mediating an antiviral immune response, and there seems to be a stronger interferon cascade in the trunk kidney.

Subsequently, KEGG pathway enrichment analysis was performed based on DEGs to further explore the similarities and differences of immune response dynamic mechanisms in the head and trunk kidney during antiviral process. Based on our results, in the head and trunk kidney at 7 dpi, most of the pathways enriched in the top were immune-related signaling pathways, particularly the TLR and NLR signaling pathways. Previous transcriptome analysis of rainbow trout head kidney after IHNV infection also showed that NLR and TLR signaling pathways were activated after infection [44]. TLR and NLR signaling pathways were further analyzed in both the head and trunk kidney, both of which belong to PRR signaling pathways. The key node genes TLR7/8 and MyD88 in the TLR signaling pathway were activated in the head and trunk kidney after IHNV infection. TLR7/8 can sense IHNV infection and recruits MyD88, which can activate various kinases (IRAK4, IRAK1) that cause the interferon regulatory factor (IRF) to promote the type I interferon response [45,46]. NLRs are PRRs similar to TLRs, and also play a crucial role in the innate immune response by recognizing PAMPs and damage-associated molecular patterns (DAMPs) [47]. NLR signaling pathways mediated in the head and trunk kidney have similar response mechanisms. Nod2 gene recognizes PAMPs (ssRNA of virus) and recruits TRAF3, followed by activation of interferon regulatory factors. However, at 28 dpi, the enriched signaling pathways were significantly different in the head and trunk kidney. In the head kidney, the cellular and molecular related signaling pathways were mostly enriched, while in the trunk kidney, the metabolic related signaling pathways were mainly enriched. This result also further indicates that the head and trunk kidney diverged in function. 

In conclusion, we successfully established an IHNV infection model of rainbow trout and detected the pathological changes and expression of immune genes in the head and trunk kidney. The gene expression and pathway enrichment in the head and trunk kidney are similar in response to IHNV infection. Additionally, although the trunk and head kidney have different tissue structures, and the function of the trunk kidney is differentiated, the trunk kidney still shows strong immune response, especially the interferon-stimulated genes, which have stronger immune response in the trunk kidney than in the head kidney. This study will provide new directions and challenges for functional research of teleost trunk kidney.

## Data Availability

Not applicable.

## Supplementary Materials

The following supporting information can be downloaded at: https://www.mdpi.com/article/10.3390/v14122663/s1, Figure S1: The IHNV standard curve and the Isotype control staining; Figure S2: Anatomical location diagram and H&E staining of head kidney and trunk kidney of rainbow trout; Figure S3: Validation of differentially expressed genes by qRT-PCR; Figure S4: Heatmap illustrates results from RNA-seq of the mRNA expression levels of genes in TLR and NLR signaling pathway; Table S1: Evaluation criteria of kidney pathological changes in rainbow trout; Table S2: Pathological scores of kidney at different time points after viral infection; Table S3: Primers used in this study.
