# Supplementary material for: Comparative Study on Immune Function of the Head and Trunk Kidney in Rainbow Trout Responding to IHNV Infection
Source: Viruses. 2022 Nov 28;14(12):2663. doi: 10.3390/v14122663 (PMC9788286; doi:10.3390/v14122663)
Supplement: Supplementary file 1 [file viruses-14-02663-s001.zip › viruses-2045168-supplementary.pdf]

Supplemental Figure S1 | (A) The IHNV standard curve was constructed. (B) Isotype control staining for IHNV antibodies in paraffin-sections of trout kidney. Scale bars, 20 μm. Data are representative of three independent experiments.

Supplemental Figure S2 | Anatomical location diagram and H&E staining of head kidney and trunk kidney of rainbow trout.

Supplemental Figure S3 | Validation of differentially expressed genes by qRT-PCR in trout head kidney (A) and trunk kidney (B).

Supplemental Figure S4 | Heatmap illustrates results from RNA-seq of the mRNA expression levels of genes in Toll-like receptor signaling pathway (A) and NOD-like receptor signaling pathway (B) in kidney of IHNV-infected fish versus control fish measured at 7 and 28 dpi (*n*=3 fish per group). Data are expressed as log2(FC). FC, fold change.

Supplemental Table S1 | Evaluation criteria of kidney pathological changes in rainbow trout.

Supplemental Table S2 | Pathological scores of kidney at different time points after viral infection (*n*=6 fish per group).

Supplemental Table S3 | Primers used in this study.

Supplemental Figure S1

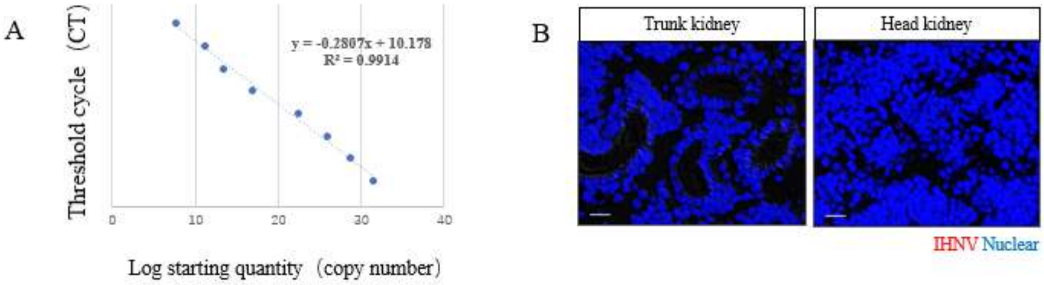

Supplemental Figure S2

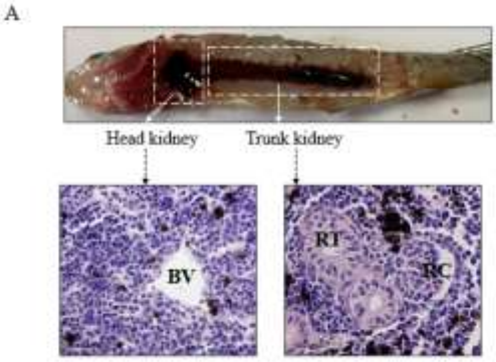

Supplemental Figure S3

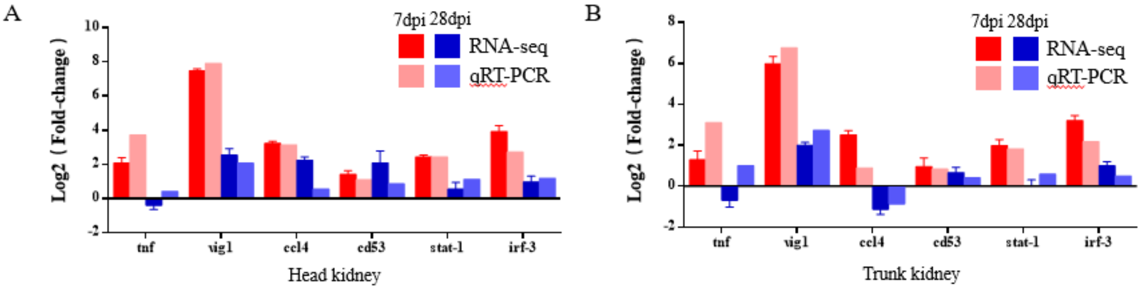

Supplemental Figure S4

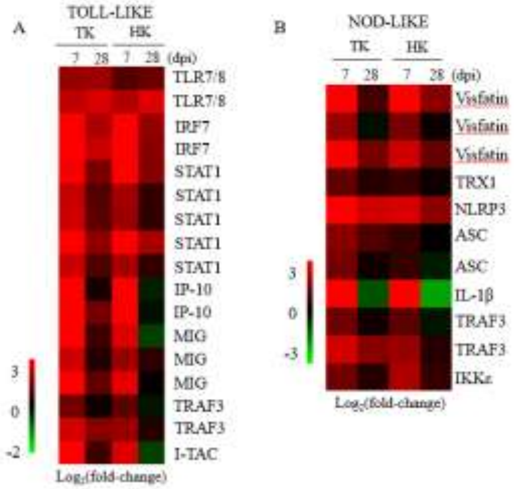

Table S1 Pathological score criteria for head kidney (HK) and trunk kidney (TK)

Pathological changes		Degree	Score
HK	**Melanin macrophages**	Normal	0
		Decrease	1
		Increase	2
	**Sinusoid**	Normal	0
		Vacuity	1
		Congestion	2
	**Blood vessel**	Normal	0
		Increased permeability	1
		Significantly increased permeability	2
	**Lymphatic cord**	Normal	0
		Disorder	1
		Disorder and lymphopenia	2
TK	**Interstitium**	None	0
		Mild	1
		Moderate	2
		Severe	3
	**Vessels**	None	0
		Mild	1
		Moderate	2
		Severe	3
	**Tubules**	None	0
		Mild	1
		Moderate	2
		Severe	3

Table S2 Pathological score of head kidney and trunk kidney

**Head kidney**	Control	4 dpi	7 dpi	14 dpi	28 dpi
Melanin macrophages	0	2	2	2	1
Sinusoid	0	2	2	2	1
Blood vessel	0	2	2	1	1
Lymphatic cord	0	2	2	2	1
**Total score (*n*=6)**	0	8	8	7	4
**Trunk kidney**	Control	4 dpi	7 dpi	14 dpi	28 dpi
**Interstitium**					
Proliferation of hematopoietic tissue	2	2	1	2	2
Necrosis	0	2	3	3	1
Hemorrhage	0	3	3	2	1
Fibrosis	0	1	0	0	0
Melanomacrophages centres	1	3	3	3	2
**Vessels**					
Thrombi	1	3	3	3	1
Necrosis of wall of vessels	1	3	2	2	1
**Tubules**					
Nephron neogenesis	3	1	1	1	3
Necrosis of renal tubules	0	3	3	2	0
Necrosis of glomerules	0	3	3	3	1
**Total score (*n*=6)**	8	24	22	21	12

Table S3 Primers used in this study

Gene	GenBank accession no.	Primer sequence (5’-3’)	
		Forward primer	Reverse primer
EF-1α	XM_021571866.1	CAACGATATCCGTCGTGGCA	ACAGCGAAACGACCAAGAGG
CXCL10	XM_021622222.1	ACATCAACGGTCCTCATC	ACACTTCTTCCCTTCTCC
CXCL9	NM_001124601.1	GTGGTTTTGCTGGGAGTTT	TTTTGCTTGTCGTCCTTGT
tnf	NM_001124374.1	CACACTGGGCTCTTCTTCGT	CAAACTGACCTTACCCCGCT
IFNAR	NM_001124531.1	CAGAGCCTCAGGAAGAACT	CAAGGGGTAGAAGAGCATA
Vig1	NM_001124253.1	AGTCTGAGGGAGGCAGAGAG	ACTGAATGGCCTCTTCCACG
LGP2	XM_036938249.1	AGTTTGGCACGCAGGAGTA	CAAGCAGGAAGAAGTCGGT
MX1	NM_001171901.1	GATGCTGCACCTCAAGTCCTACTA	CGGATCACCATGGGAATCTGA
CCL4	NM_001124489.2	CCCTCGTCTTGCTATGCTGT	ACAGAATGGCTGGGTTGGAG
RIG-1	AGN48009.1	CAGAGGTACTACAGGAAATGG	TTACTGGTCTTCAAGCAATG
IL-8	NM_001124362.1	TGTCGTTGTGCTCCTGG	CCTGACCGCTCTTGCTC
TRIM25	XM_021557233.2	AAAGATTCACCCCAAAACC	AAGGCAGGGGAATCATAGT
CATH1	NM_001124480.1	CTGGAGGCAAGCAACAAC	CCCCCAAGACGAGAGACA
IgT	AY870264	CAGACAACAGCACCTCACCTA	GAGTCAATAAGAAGACACAACGA
IgM	OMU04616	AAGAAAGCCTACAAGAGGGAGA	CGTCAACAAGCCAAGCCACTA
IgD	JN173049.1	CAGGAGGAAAGTTCGGCATCA	CCTCAAGGAGCTCTGGTTTGGA
CD53	NM_001165182.1	GTGTGGAACGGCAGTGTTTG	GGAGGCAGCGGTTCTCTTTA
STAT1	XM_021621820.2	GTCATCTCCAACGTCAGCCA	CAGGAGGGCTCAGGAAGAAC
IRF-3	NM_001257262.1	CAAACCGCTCCTCATCCCAT	ACCATTGCTAACCTCAGCCC
CCL19	XM_021622215.2	GTTTCCCTCGCCACTTCAA	GCCACCCACTTGCTCTTTG
TNFα	NM_001124357.1	CAGAGCCTCAGGAAGAACT	CAAGGGGTAGAAGAGCATA
IL-2	NM_001164065.2	TGTCTACAAGGAAACCCAA	GCTGCAACAATGCAACTAT
C3	XM_021568201.2	CCTCACAACAAGAGTGCACATC	CCAAGTGGGCAAACTCATCTCC
